# The Treatment of Acute Maniacal Delerium

**Published:** 1893-12-09

**Authors:** Cecil F. Beadles

**Affiliations:** Assistant Medical Officer, Colney Hatch Asylum


### The Hospital Clinic.

[*The Editor will be glad to receive offers of co-operation and contributions from members of the profession. All letters should be addressed to* The Editor, The Lodge, Porchester Square, London, W.]

### THE TREATMENT OF ACUTE MANIACAL DELERIUM.

### Means Directed to Relieve the Acute Symptoms and the Subsequent Treatment.

By Cecil F. Beadles, M.R.C.S., L.R.C.R., Assistant Medical Officer, Colney Hatch Asylum.

(*Concluded from page* 138.)

The hydrobromate of hyoscine is a drug that has very similar action to hyoscyamine, but its dose is very different, being $\frac{1}{200}$ to $\frac{1}{60}$ grain. Personally I never use hyoscine, for owing to its similarity in name to hyoscyamine and the great difference in strength, these two drugs in dispensing might very readily be confused and give rise to disastrous results.

At the onset of an acute attack of the nature we are now considering, the bowels will more often than not be found to be constipated, not having acted for several days, it may be a week or even more. Especially is this so with women. It is therefore advisable to give a brisk purge in the form of Epsom salts or a couple of minims of croton oil on sugar. If the patient cannot be induced to take it, the latter may be placed in the food, but this is a plan never to be recommended, as it is apt to give rise to the idea that the food is poisoned. The chances are that he will refuse it in consequence, and thus we have to deal with an additional trouble in the treatment of the case.

Although it is somewhat unusual for these patients to entirely refuse their food, yet it may be necessary to have recourse to feeding. If the attendant or nurse in charge is unable to feed with the spoon, it is better at once to take to the feeding tube. For this purpose the oesophogeal tube is perhaps the most convenient. It may be necessary to fasten the patient in the chair, but this may frequently be avoided if there is plenty of help at hand. Some difficulty may be experienced in passing the tube, as the patient may with great muscular power force the larynx up and bring the fauces together, but this can usually be overcome by carefully guiding the oiled tube along the roof of the mouth, and over the epiglottis with the left index finger. The reaching of the patient and the attempt to force the fluid back may be frustrated by having an attendant standing behind the patient's head, to hold the gag in the mouth with his left hand, and close the nostrils with his right. The patient then breathes through his mouth, and usually takes his feeding quietly. If the bowels have not been recently opened, it may be as well to administer a full dose of Epsom salts at the same time, and a dose of tonic medicine, if it is thought necessary, may be given through the tube with the food.

A thorough cleansing of the primæ viae may be the turning point to recovery. The delirium may subside, the patient may fall into a sound sleep, from which he awakes restored in mind.

The wet-pack that was once so extensively employed to procure sleep, and is still used to a certain extent in medical practice, has now fallen into disuse in asylums. This is doubtless due to the fear that it will be considered by the Commissioners in Lunacy as a form of restraint. This is to be regretted, for I have no doubt that in many cases, if used early, it might be employed with advantage to the patient, and in many cases would seem preferable to dosing with some powerful drug. There are cases in which warm baths at a temperature of about 98 deg. can be used with distinct advantage, both as a sedative and a soporific.

In acute maniacal delirium the prognosis depends greatly on the extent to which the patient takes his food. If he takes a fair amount of nourishment, then there is every hope of an early recovery; but supposing he obstinately refuses all that is set before him, and he cannot be induced to take anything, then the prognosis becomes more grave. It is most important, therefore, to attend to the diet from the first, and to see that the patient is taking a sufficient amount. Food requires to be of a nourishing kind, and should be of a solid nature so long as the patient can be coaxed to take it. It usually happens, however, that sooner or later this fails, and we must then feed with a spoon on slops and liquids. Beef tea, concentrated extracts, milk with eggs beaten up, are required, and it may be advisable to give brandy in addition; but stimulants should not be pushed while excitement continues. Malt liquors may, however, be recommended.

The acute attack is rapidly followed by great exhaustion, in which the patient lies in a helpless state, and in spite of taking a fair amount of nourishment, if the excitement has been excessive and prolonged, the patient rapidly emaciates, and may sink into a typhoid condition. The pulse is feeble, the tongue furred, and the breath and skin have an offensive odour. Urine and motions are passed involuntarily. There will now be great difficulty in rallying the patient, notwithstanding the administration of brandy at frequent intervals. Artificial feeding should not be postponed until such a condition has set in. Nutrient enemata appear practically useless in the insane, apart from the difficulty in giving them, and are best entirely avoided. I have never known a life saved by their means. If the stage above described has been reached, the case becomes almost a hopeless one, for it is seldom that recovery takes place; the patient passes into a state of coma, and death occurs from sheer exhaustion. Such a result it is at times impossible to avoid.

If, however, a moderate amount of sleep has been obtained, we may look forward to an early recovery. This will gradually set in and proceed apace. Nourishing food must be persevered in, and be of a wholesome and varied nature. As the patient regains strength he may be put on a tonic mixture, such as quinine and iron, if he has not already been taking one, and cod liver oil may be added. In the case of a female, the catamenia should be attended to and promoted, for there will usually be found to be amenorrhœa present. Plenty of fresh air should be allowed, and the patient, if able, may take exercise out of doors, and some useful employment should be provided by which to occupy the attention. All such means greatly tend to promote the general health, and towards a rapid recovery.

